# The Relationship Between Reactive Oxygen Species and Endothelial Cell Metabolism

**DOI:** 10.3389/fchem.2020.592688

**Published:** 2020-11-26

**Authors:** Raid Alhayaza, Emaan Haque, Catherine Karbasiafshar, Frank W. Sellke, M. Ruhul Abid

**Affiliations:** ^1^Alfaisal University School of Medicine, Riyadh, Saudi Arabia; ^2^Division of Cardiothoracic Surgery, Cardiovascular Research Center, Rhode Island Hospital, Brown University Alpert Medical School, Providence, RI, United States

**Keywords:** reactive oxygen species (ROS), endothelial cell metabolism, angiogenesis, mitochondria, nictonamide adenine dinucleotide phosphate (NADPH) oxidase, oxidative phosphorylation, glycolysis, fatty acid oxidation

## Abstract

Cardiovascular disease (CVD) has been the leading cause of death for many decades, highlighting the importance of new research and treatments in the field. The role of hypoxia and subsequent free radical production [reactive oxygen species (ROS)] have become an area of particular interest in CVD. Interestingly, our laboratory and other laboratories have recently reported positive roles of subcellular ROS in modulating endothelial cell (EC) metabolism, proliferation, and angiogenesis. This bidirectional relationship between ROS and EC metabolism, as well as functional changes, continues to be an area of active research. Interestingly, ECs have been shown to rely on anaerobic processes for ATP generation, despite their direct access to oxygen. This paradox has proven to be beneficial as the major reliance on glycolysis produces ATP faster, preserves oxygen, and results in reduced ROS levels in contrast to oxidative phosphorylation. This review will address the relationship between ROS and carbohydrate, lipid, and nitrogen metabolism in ECs, and their effects on EC phenotype such as sprouting angiogenesis.

## Introduction

Cardiovascular diseases (CVDs) are considered to be a major public health burden and a leading cause of global morbidity and mortality (Lozano et al., 2012; Murray et al., 2012). The elevation of reactive oxygen species (ROS) levels has been linked to various forms of CVDs (Ying et al., 2004). Endothelial dysfunction is triggered by oxidative stress and is associated with many CVD stressors and risk factors, making it the hallmark of CVDs and an early predictor of atherosclerosis. Endothelial cells (ECs) rely on endothelial nitric oxide synthase (eNOS) not only for its regular function and activity but also for the vasculature integrity and homeostasis as a whole. However, the eNOS activity can be lost or even the enzyme can be uncoupled by oxidative stress (Daiber et al., 2019). Oxidative stress also increases the permeability of vascular ECs, promotes the adhesion of leukocyte, and alters EC signal transduction (Lum and Roebuck, 2001; Abid et al., 2007; Lee et al., 2010).

Despite the fact that ROS are being generated at injured and inflamed sites, it has been shown that low levels of ROS have an important regulatory role as signaling intermediates that are essential for different cell activities such as cell adaptation and growth (Abid et al., 2000, 2001, 2006, 2007). On the other hand, high ROS levels can result in cell injury and death. Thus, ROS have a paradoxical effects on EC metabolism (Lum and Roebuck, 2001). This review discusses the relationship between ROS with carbohydrate, lipid, and nitrogen metabolism in ECs.

## Background and Physiological Relevance

### ROS and Reactive Nitrogen Species

**Figure 1:**
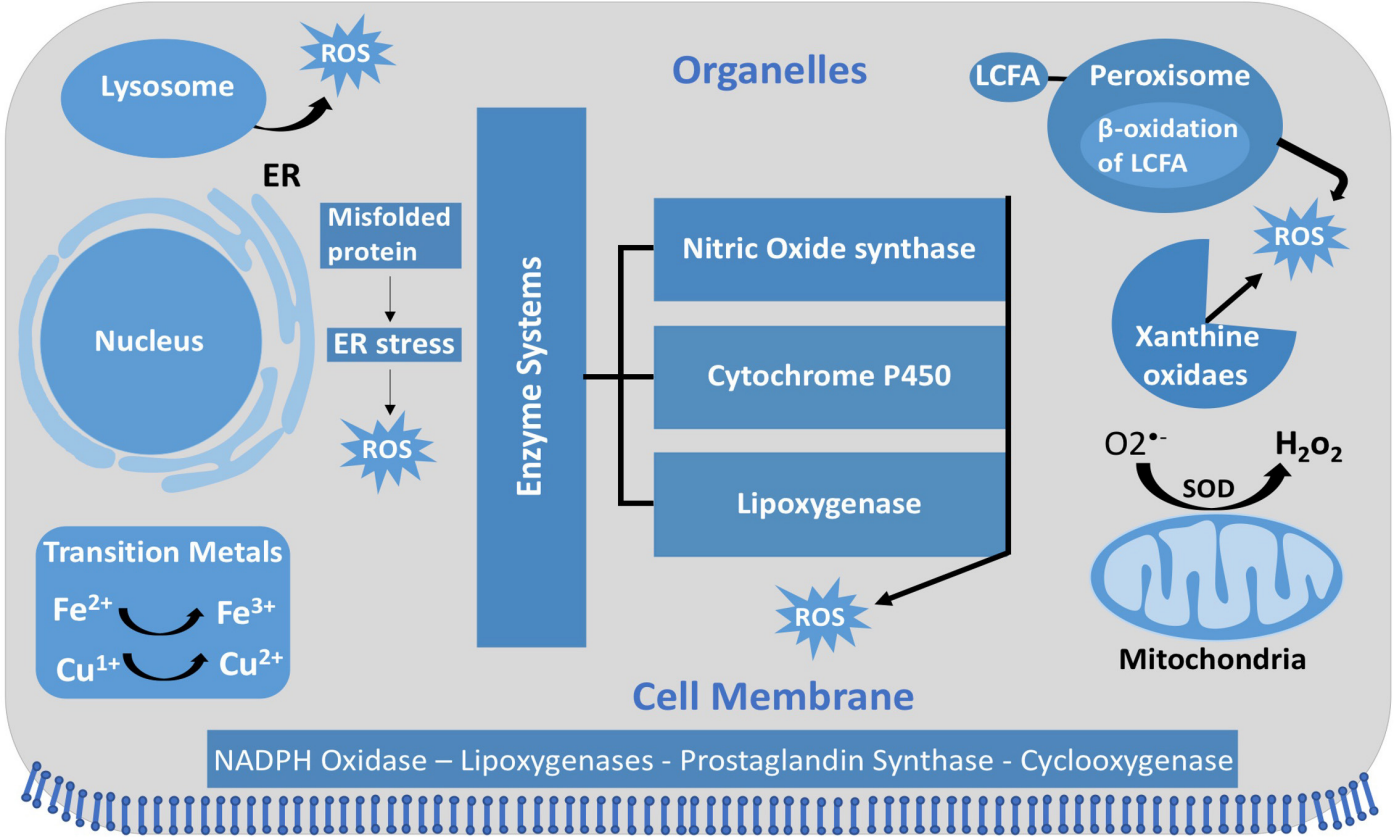
Intracellular ROS and their sources and localization in EC. In addition to NOX, intracellular sources of ROS include lysosome, ER, peroxisome, and mitochondria. SOD, superoxide dismutase; GSH, glutathione; ER, endoplasmic reticulum; LCFA, long-chain fatty acid.

**Figure 2:**
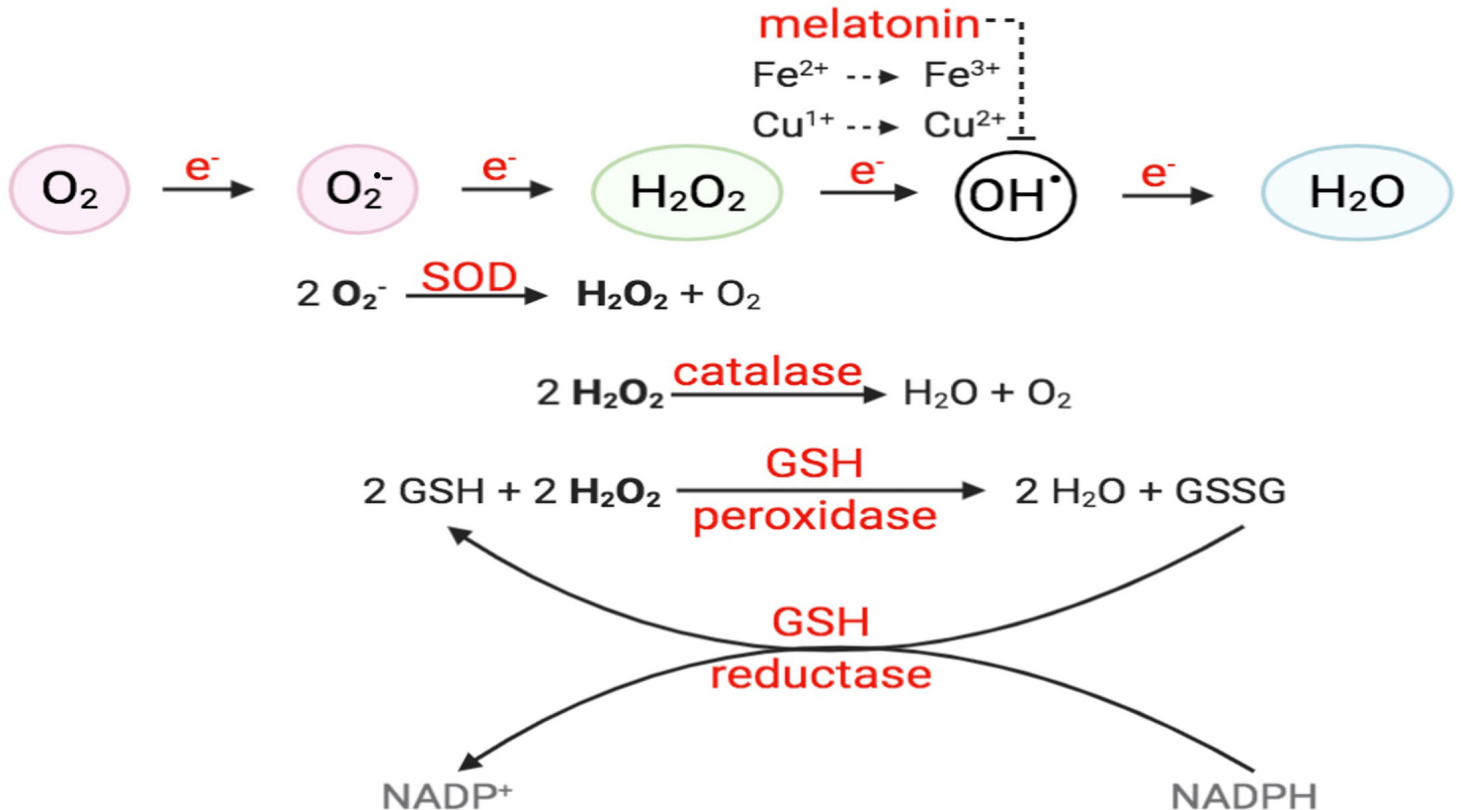
O_2_ reduction process and antioxidant enzymes. For O_2_ to be fully reduced and stable, it must accept four electrons in the eventual formation of water. Failure to do so results in the generation of free radicals. O_2_ with the addition of one electron forms superoxide anion (${\text{O}}_{2}^{•-}$); O_2_ + two electrons will form hydrogen peroxide (H_2_O_2_). O_2_ + three electrons will form hydroxyl radical (^•^OH), in the presence of transition metals, like Fe^2+^ and Cu^+^. Intracellular enzymes that work as antioxidants include SOD, catalase, and GSH peroxidase and reductase. Melatonin has also been shown to decrease hydroxyl radical formation.

ROS are molecules with unpaired electrons in their outer orbit. These unpaired electrons give oxygen species a high degree of reactivity, which can lead to damaging effects. ROS stabilize themselves by stealing electrons from other molecules. Interestingly, ROS are formed both physiologically and pathologically (Figure 1). In the physiological sense, ROS form through metabolic processes, predominantly oxidative phosphorylation where cytochrome c oxidase transfers electrons to oxygen molecules (O_2_). Cellular antioxidants defend against ROS by participating in the O_2_ reduction process, in which O_2_ must accept four electrons in order to be stable, to be fully reduced, and to produce water. However, if it fails to receive all four electrons and was only partially reduced, this outcome will generate free radicals resulting in oxidative stress, or an imbalance between the formation of ROS and antioxidants (Figure 2). Moreover, ROS include superoxide anion (${\text{O}}_{2}^{•-}$), hydrogen peroxide (H_2_O_2_), and hydroxyl radical (^•^OH), which are sequentially arranged by the number of electrons they acquire. H_2_O_2_ has paired electrons but is still considered reactive and can aid in hydroxyl radical (HO^•^) generation, which is the most reactive and toxic ROS. Hydroxyl radicals are formed in the presence of H_2_O_2_ and a transition metal, like copper (II) and iron (II), which induces a Fenton-like system (Day, 2009; Craige et al., 2011; Schröder et al., 2012; Shafique et al., 2013; Hojo et al., 2017; Kim et al., 2017; Aldosari et al., 2018; Bodega et al., 2019; Wang et al., 2019).

**Figure 3:**
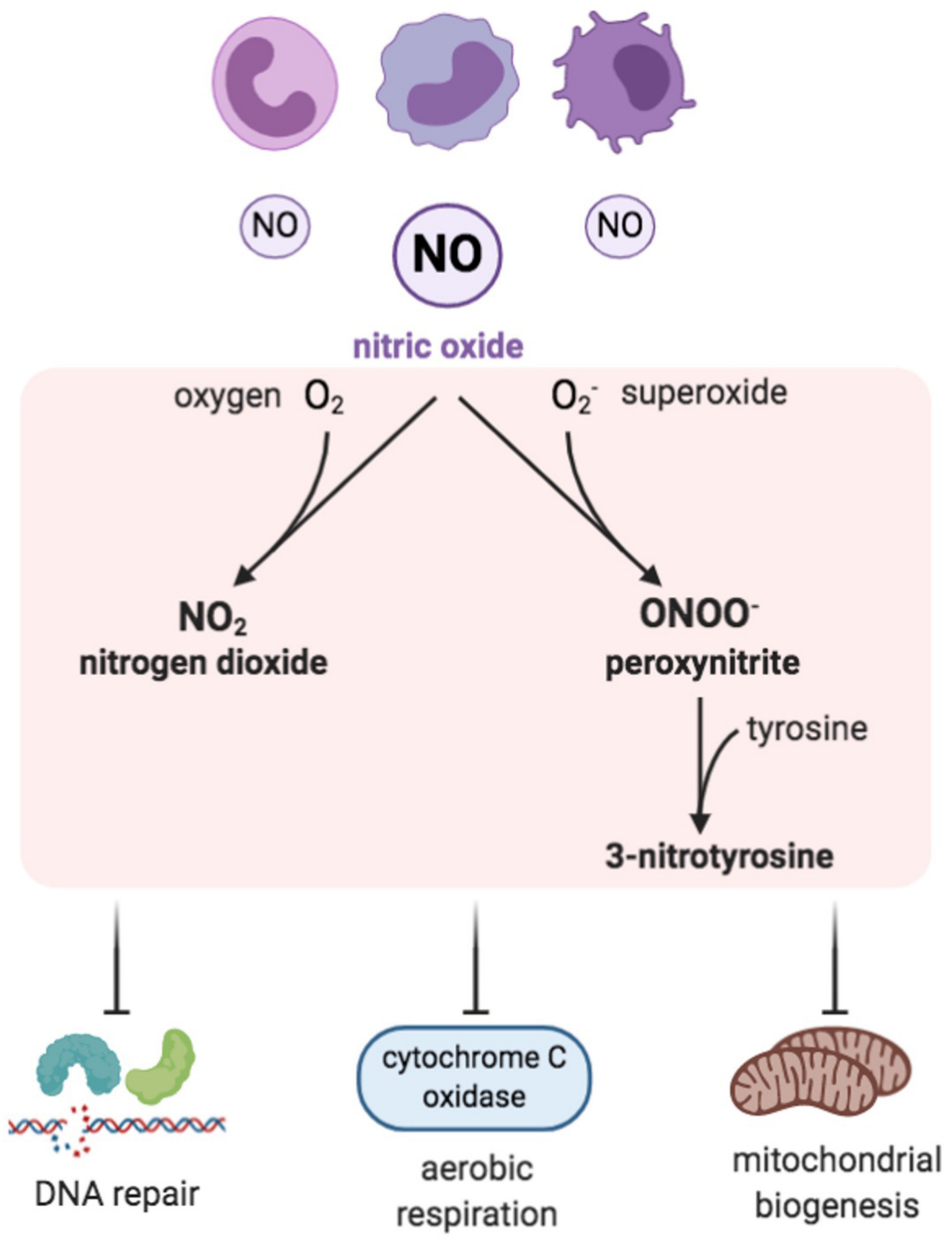
Reactive nitrogen species (RNS). Inflammatory cells are a prominent source of nitric oxide and, when in excess, RNS are formed by combining NO with ROS, especially with superoxide. Examples of RNS include peroxynitrite, nitrogen dioxide, and 3-nitrotyrosine, which have inhibitory effects on DNA repair enzymes, cytochrome c oxidase, and mitochondrial biogenesis.

The term reactive oxygen nitrogen species (RONS) collectively describes two types of free radicals, which are the reactive oxygen and nitrogen species. RONS are formed in response to stressful and inflammatory situations and are released from phagocytes, such as neutrophils, macrophages, and dendritic cells (Salman and Ashraf, 2015). RONS are produced by a variety of endogenous organelles (Figure 3) (Giustarini et al., 2009); in fact, one of the major sources of RONS is the mitochondria. Surprisingly, these reactive species in turn regulate mitochondrial activity through different mechanisms and pathways, such as lipid peroxidation, mitochondrial biogenesis, mtDNA damage, and mitochondrial membrane permeability transition pore (Nisoli et al., 2003, 2004; Ma, 2011; Piantadosi and Suliman, 2012). RONS are extremely unstable and reactive due to their unpaired electrons and non-static bonds, which require an appropriate control and regulation to prevent any associated tissue damage and immune system disturbance (Salman and Ashraf, 2015).

Reactive nitrogen species (RNS) are nitrogen-containing oxidants or, in other words, nitrogen-centered free radicals that have an essential and a direct role in immune responses, vasodilatation, and cellular signaling (Drew and Leeuwenburgh, 2002). RNS consist of nitric oxide (NO.) and its by-products, which include peroxynitrite (ONOO^−^.), nitrogen dioxide (NO_2_.), and 3-nitrotyrosine (Figure 3) (Klebanoff, 1980; Bedard and Krause, 2007). RNS can be generally classified as non-ions [nitric oxide (NO^•^)] or as ions [peroxynitrite (ONOO^−^.)]. Nitric oxide is generated by the mitochondria through nitric oxide synthases (NOSs), which are a family of NADPH-dependent enzymes. Various isoforms of NOS have been identified—neuronal isoform (NOS1), inducible isoform (NOS2), and endothelial isoform (NOS3), in addition to the mitochondrial isoform (mtNOS) (Ghafourifar and Richter, 1997). NOS catalyzes the breakdown reaction of arginine to citrulline, generating nitric oxide (NO) (Bolisetty and Jaimes, 2013).

NO is a neurotransmitter and a regulator of blood pressure. It is capable of generating potent oxidants during pathological states (Salman and Ashraf, 2015). Although NO is not a potent reactive free radical by itself, it has the ability to generate other reactive intermediates, which, in turn, affects the organism in its entirety and the protein function in particular. These reactive intermediates can result in deleterious effects by triggering biomolecule nitrosative damage (Drew and Leeuwenburgh, 2002). The NO directly alters the DNA by inhibiting the DNA repair enzymes (Salman and Ashraf, 2015). NO excess is linked to a group of medical conditions, such as neuro-degenerative diseases, ischemic reperfusion injuries, and chronic inflammatory diseases like inflammatory bowel disease and rheumatoid arthritis (Sharma et al., 2007). NO synthesis can lead to cytochrome c oxidase suppression (Figure 3). Furthermore, mitochondrial nitric oxide increases RNS and ROS production, which subsequently alters mitochondrial biogenesis and oxidative stress (Kong et al., 2010; Bolisetty and Jaimes, 2013). Moreover, NO can bind to heme groups in the proteins of the electron transport chain, including cytochrome c oxidase, which results in inhibition of respiration (Poderoso et al., 1996; Mason et al., 2006).

#### ROS Generation

**Figure 4:**
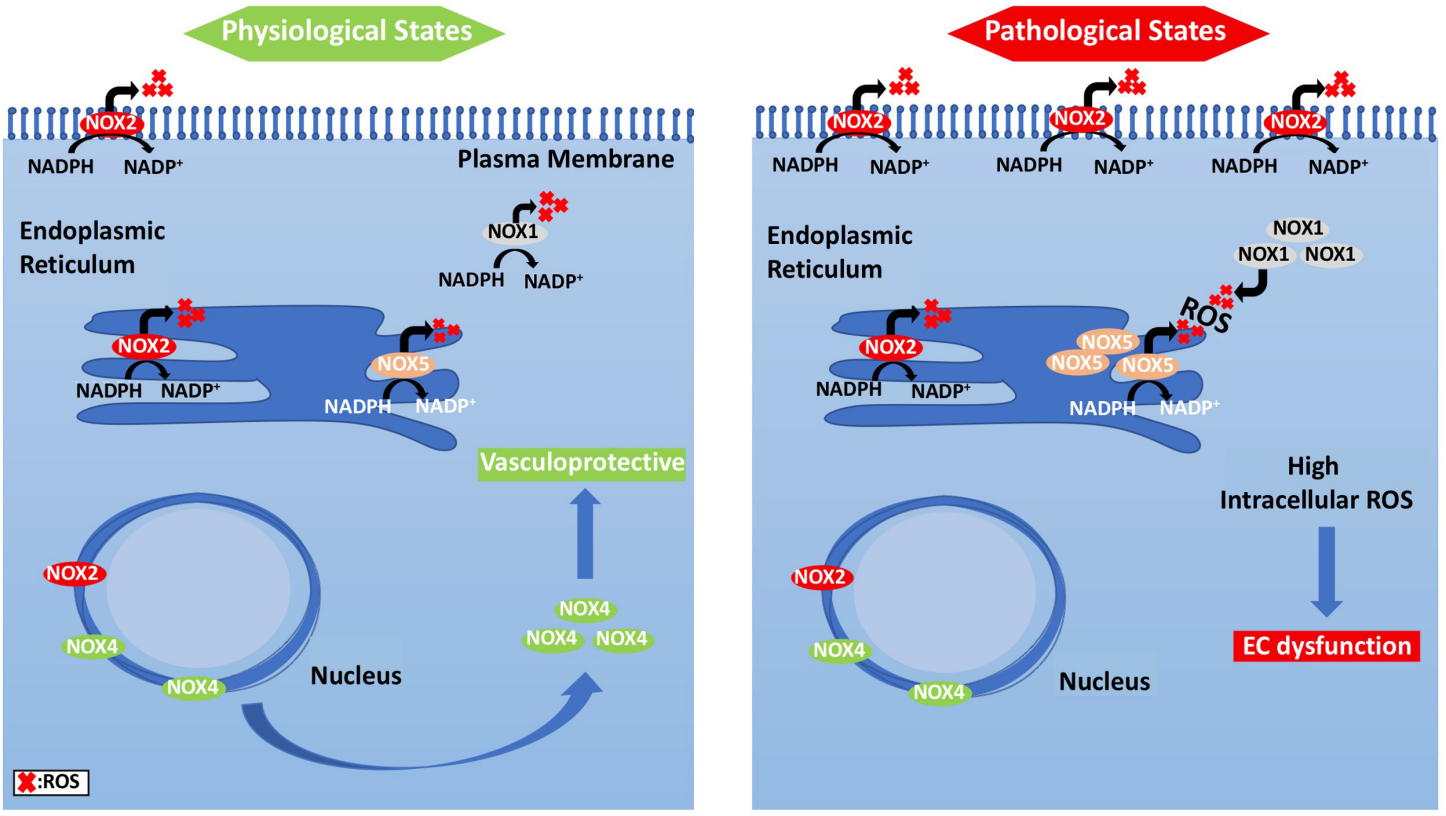
Subcellular localization of NADPH oxidases and compartmentalization of NOX2-derived ROS and their effects on EC signaling pathways. **(Left panel)** ECs under physiological states, where NOX2 expression is low and NOX4 levels are increased, show vasculo-protective phenotype. **(Right panel)** Under pathological states, however, NOX2 forms clusters, and is responsible for ROS generation.

Unlike other cells, EC mitochondria are not considered as the primary source of ROS generation, which could be associated with the small percentage of mitochondria to EC mass (Aldosari et al., 2018). Instead, nicotinamide adenine dinucleotide phosphate (NADPH) oxidases are considered to be the main source of ROS formation in ECs; additional sources include the lysosome, peroxisome, and endoplasmic reticulum (ER) (Craige et al., 2011; Schröder et al., 2012; Shafique et al., 2013; Kim et al., 2017). NADPH oxidase is composed of five subunits; p47^phox^, p67^phox^, and Rac1 are the regulatory cytoplasmic subunits that possess GTPase activity, while Nox2 (gp91^phox^) and p22^phox^ are membrane bound (Feng et al., 2010). The membrane-bound NOX (NADPH oxidase) has multiple isoforms that contribute to ROS production. Those most important in the vasculature are NOX1, NOX2 (gp91^phox^), NOX4, and NOX5, which exist in multiple subcellular organelle and/or membranous compartments in ECs, such as the perinuclear membrane, ER, and cell membrane (Bayraktutan et al., 2000; Van Buul et al., 2005). Moreover, NOX2 and NOX4 are found in the cell membrane and shown to have important roles in influencing cell signaling and cellular functions (Chen et al., 2012; Shafique et al., 2017). Under physiological states, NOX2 is expressed in low levels in the nuclear membrane and ER to participate in the redox signaling pathways intracellularly. On the other hand, clusters of NOX2 in the plasma membrane were noticed under disease conditions that resulted in increased superoxide production extracellularly (Figure 4) (Drummond and Sobey, 2014). Moreover, NOX-derived ROS are shown to be essential in VEGF signaling and the activation of eNOS pathway, which results in nitric oxide synthesis and the vasodilation of blood vessels (Abid et al., 2000, 2006, 2007; Lee and Griendling, 2008; Feng et al., 2010).

#### ROS Sensors

When ECs face stressful situations, such as hypoxia and fluid shear stress, they produce ROS, which then regulate the pro-survival kinase AMPK (Shafique et al., 2013), known to be activated during the reduction of AMP/ATP ratio and starvation (Mungai et al., 2011; Wang et al., 2011; Mackenzie et al., 2013). AMP-activated protein kinase (AMPK) is important for cellular redox signaling (Shafique et al., 2017), metabolic adaptation, and detecting energy-deprived states such as hypoxia (Lee et al., 2003). AMPK has an essential role in regulating several transcription factors and signaling intermediates that include hypoxia-inducible factor 1-alpha (HIF-1α), PPAR gamma co-activator 1 alpha (PGC-1α), and Forkhead box protein O1 (FOXO1) (Lee et al., 2003; Dixit et al., 2008; Aatsinki et al., 2014; Awad et al., 2014; Hwang et al., 2014; Wan et al., 2014; Yun et al., 2014). HIF-1α is a transcription factor that induces gene expression of erythropoietin, glucose transporters, VEGF, etc. to support erythropoiesis and angiogenesis (Lee et al., 2003). PGC-1α is a transcriptional co-activator that regulates energy metabolism and mitochondrial biogenesis (Wan et al., 2014) to improve EC survival and proliferation.

**Figure 5:**
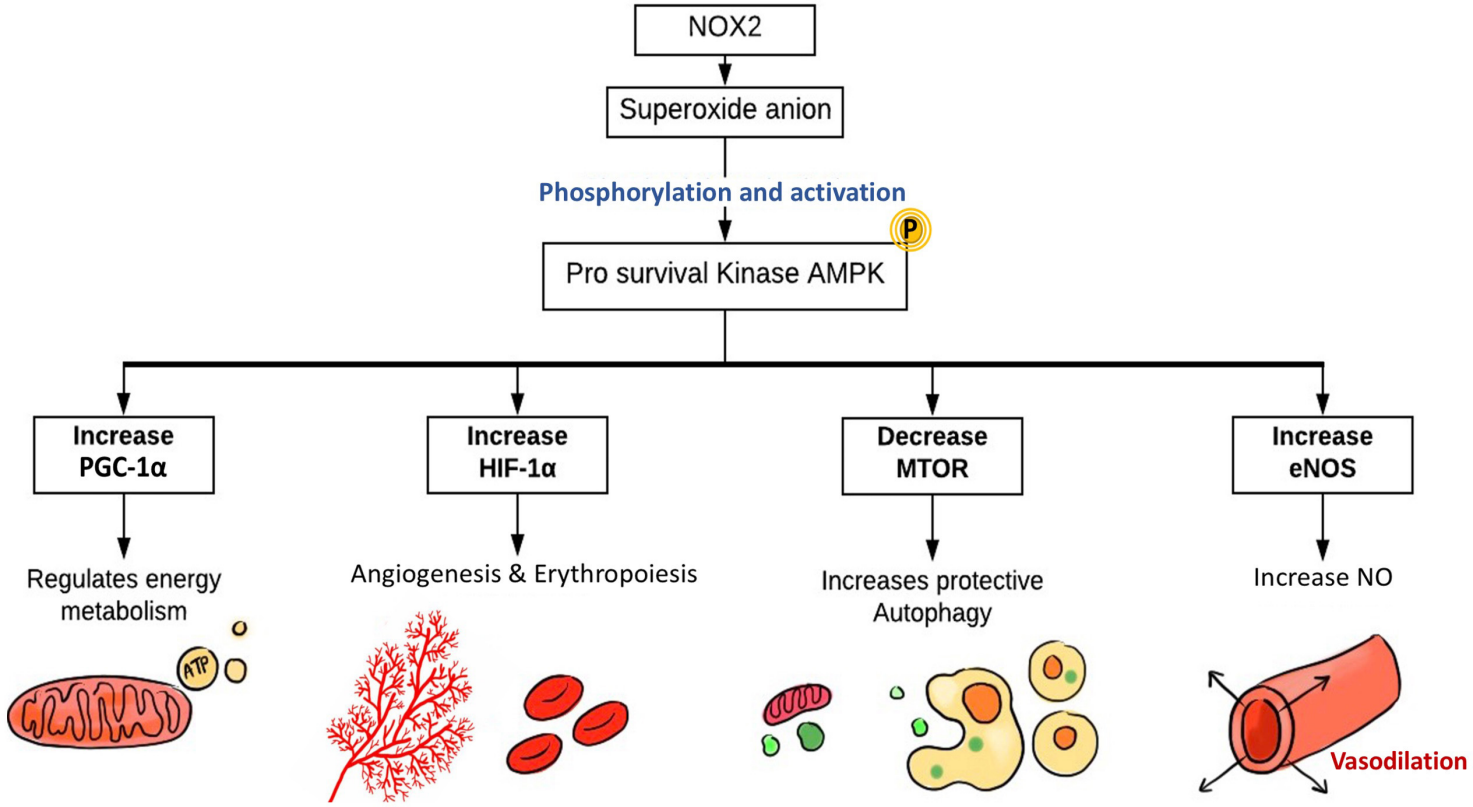
The role of EC-specific Nox2-derived ROS in enhancing ECs function. The main source of ROS in ECs are NOX, particularly NOX2, which generates superoxide. NOX-derived ROS activate AMPK, PGC1-α, and HIF-1α. The pro-survival kinase AMPK increases eNOS activation and NO synthesis resulting in coronary vasodilatation. HIF-1α expression induces angiogenesis and erythropoiesis, while PGC1-α regulates mitochondrial biogenesis, energy metabolism, and protective autophagy. NOX, NADPH oxidase; AMPK, AMP kinase; PGC-1α, PPARG coactivator 1 α; HIF-1^α^, hypoxia-inducible factor 1α; mTOR, mammalian target of rapamycin; eNOS, endothelial nitric oxide synthase.

The protective role of AMPK is further established through its part in autophagy regulation, a process that helps in reutilizing organelles and macromolecules that have been damaged through the lysosomal degradation pathway (Kimura et al., 2007; Wang et al., 2011; Cardaci et al., 2012; Li et al., 2014; She et al., 2014; Weerasekara et al., 2014). The activation of AMPK is linked to caloric restriction states where consumption of energy is reduced due to nutrition deprivation (Frank W Sellke, 2014). High redox levels in ECs are believed to activate AMPK through Ca^2+^/calmodulin-dependent protein kinase kinase-β (CaMKKβ)-dependent pathway (Shafique et al., 2013). AMPK has an inhibitory effect on the mammalian target of rapamycin (mTOR) pathway, which results in induction of protective autophagy. Additionally, the vasodilatory effect of AMPK is mediated by eNOS, eventually leading to NO synthesis and endothelium-dependent coronary vasodilatation (Figure 5) (Shafique et al., 2013).

#### Antioxidant Defenses

EC has several defense mechanisms, which include various enzymes that act as antioxidants. Superoxide dismutase (SOD) catalyzes the conversion of two ${\text{O}}_{2}^{•-}$ to water (H_2_O) and oxygen (O_2_), while catalase converts two H_2_O_2_ molecules to O_2_ and H_2_O. H_2_O_2_ can also react with two glutathiones (GSH) and will result in two H_2_O and glutathione disulfide (GSSG). Moreover, glutathione reductase uses reducing power derived from β-NADPH to convert GSSG to two GSH (Day, 2009). On the other hand, hydroxyl radicals are neutralized via melatonin, which acts as a strong pro-oxidant in the Fenton-like system, likely attributed to its ability to reduce copper (Figure 2) (Mayo et al., 2003; Wang et al., 2019). Additionally, recent studies have highlighted the significance of thioredoxins in facilitating the reduction of ROS by cysteine thio-disulfide exchange, hence protecting protein structures against oxidative stress (Tanaka et al., 2020).

Enzymatic antioxidants are upregulated in stressful situations, like oxidative stress, a lot of which are regulated by the transcription factor nuclear factor erythroid 2-related factor 2 (Nrf2). In normal physiological state, Nrf2 is commonly linked with a repressor protein known as Keap-1. This link results in a low cytoplasmic Nrf2 level. ROS and other stressors will aid in Nrf2 accumulation and translocation to the nucleus. This will favor the binding of Nrf2 to a specific element in the genes coding for antioxidant proteins, which is the antioxidant response element. On the other hand, low levels of Nrf2 will favor oxidative stress, and it might contribute to the pathophysiology of a lot of diseases such as diabetes mellitus, atherosclerosis, obesity, and cardiovascular disorders (Da Costa et al., 2019). ROS have been linked to many pathological conditions including CVDs. This connection was due to the high level of ROS concurrently witnessed with CVDs, such as ischemic heart disease and coronary artery disease (Frey et al., 2009; Datla and Griendling, 2010; Lassègue and Griendling, 2010; Luo et al., 2010; Fukai and Ushio-Fukai, 2011). The notion of ROS being harmful can be attributed to our basic recognition of their bactericidal effects in phagocytes, not considering the different sources of ROS and various research results of ROS induction by non-specific chemicals (Bayraktutan et al., 2000; Abid et al., 2001; Ushio-Fukai et al., 2002). All of these have led to a perception that improving cardiovascular function can be achieved through ROS reduction. Interestingly, many clinical trials have failed to reduce cardiovascular morbidity and mortality when using antioxidants to decrease ROS levels [e.g., Heart Outcomes Prevention Evaluation (HOPE) and Alpha-Tocopherol Beta-Carotene (ATBC) (Rapola, 1996; Rapola et al., 1998; Virtamo et al., 1998; Hegele, 2000; Vivekananthan et al., 2003; Willcox et al., 2008)]. These unexpected findings may be attributed to the important roles of ROS in signal transduction, including nitric oxide synthesis and vasodilation (Sawada et al., 2008; Zhang et al., 2008; Feng et al., 2010).

Mitochondrial-derived ROS also play an important role in endothelium-dependent vasodilation. When ECs are exposed to risk factors associated with cardiovascular risk, the endothelial mitochondria generate elevated levels of ROS, which serve as essential signaling molecules that activate pro-inflammatory and prothrombotic pathways in the vascular endothelium. Surprisingly, this process initially manifests as endothelial dysfunction and, in the long term, may result in atherosclerotic plaque formation (Widlansky and Gutterman, 2011).

### EC Metabolism

The endothelium is often overlooked as one of the most remarkable organs in the body. It is also considered to be one of the largest and most heterogeneous organs, comprising the lining of extensive vasculature throughout the body, where they facilitate the delivery of oxygen and nutrients (Fitzgerald et al., 2018). It was initially thought that ECs are solely responsible for guiding blood; however, ECs have been shown to play an essential role in health and vascular diseases; it serves many key and unparalleled functions without which human life would not be possible. In embryological life, endothelial vessels are derived from the mesodermal germ layer. Some mesoderm cells differentiate to hemangioblasts, which can further differentiate to angioblasts that subsequently form ECs. Moreover, the endothelium consists of many cellular subtypes with different locations, functions, and phenotypes. Vascular endothelial growth factor (VEGF) is the main driving force for the designation of ECs into a specific subtype. This factor is produced in hypoxic tissue to attract new vessel sprouts to replenish oxygen and nutrient supply (Eelen et al., 2015). EC metabolism is highly regulated and critical for meeting the cells' high demand of biosynthesis, redox, and energy. Lymphatic, venous, microvascular, and arterial ECs each have unique functions, core metabolism, and oxygen consumption (Eelen et al., 2015). For example, the levels of intracellular ATP, oxygen, and glucose consumption vary between pulmonary arterial ECs and pulmonary microvascular ECs (Parra-Bonilla et al., 2010). Furthermore, it has been found that mitochondria are significantly more present in brain microvascular ECs compared to ECs located in the periphery (Tang et al., 2014).

VEGF-Delta like ligand 4 (Dll4)-Notch signaling cascade is important for the process that labels an EC as a tip cell and for natural vascular remodeling (Leslie et al., 2007). Traditionally, VEGF-Dll4-Notch signaling cascade was perceived as the only process governing the transition from the EC quiescent state to angiogenic state. However, recent scientific research now recognizes a metabolic switch that must be accompanied with the VEGF-Dll4-Notch signaling cascade. These metabolic pathways—involving glucose, fatty acids (FAs), and glutamine and working through enzymes such as phosphofructo-2-kinase/fructose-2,6-bisphosphate (PFKFB3), carnitine palmitoyltransferase 1a (CPT1a), and glutaminase 1 (GLS1)—were previously unknown to have a role in EC proliferation, but are now believed to have a critical role in promoting angiogenesis. A study done by Kuosmanen et al. showed that NRF2 together with miR-93 control the switch of EC from the quiescent to the active state and vice versa (Kuosmanen et al., 2018). It is also of significance to note the heterogenicity of ECs throughout the body, especially the differences that arise through organ specificity and pathological conditions such as tumor growth. According to the type of organ in which they are present, ECs can differ in the capillary permeability (continuous, fenestrated, and sinusoidal), angiogenic potential, and metabolism (Marcu et al., 2018). We will shed light on EC metabolism by focusing on the various metabolic pathways and their role in vessel sprouting—particularly amino acid (AA), glucose, and fatty acid metabolism, which are considered to be the three major energy sources.

### Angiogenesis

The formation of new blood vessels is a complex process that plays an important role in both physiological and pathological conditions. Angiogenesis and vasculogenesis are two distinct ways to form a new blood vessel (Patan, 2010). Angiogenesis is the development of new blood vessels from an existing vasculature, while vasculogenesis is the process of forming new vasculature by a *de novo* production of ECs. Traditionally, it was thought that the process of vasculogenesis was only present in embryonic life but recent evidence suggests that it can also take place in adult bone marrow and vascular circulation.

**Figure 6:**
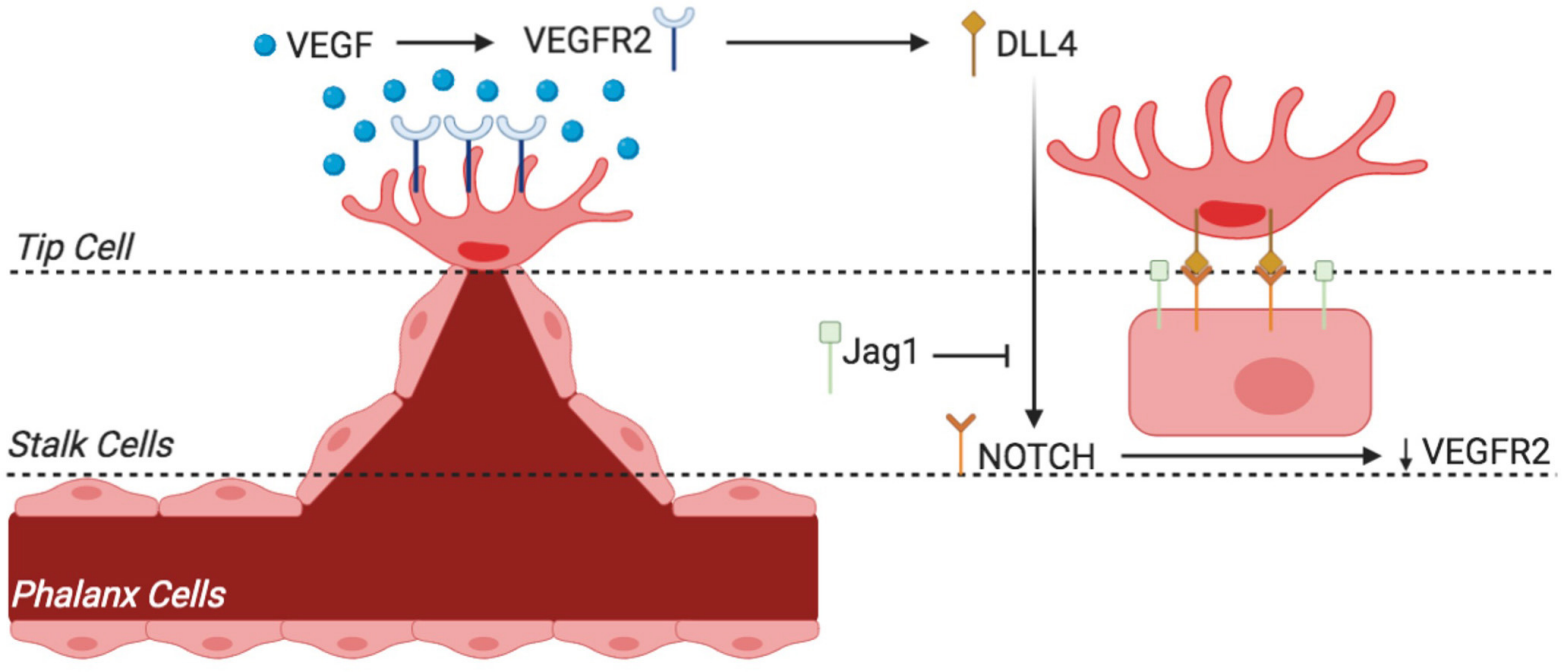
Sprouting angiogenesis and phenotypic switching of tip vs. stalk endothelial cell (EC) by VEGF-Notch signaling. The endothelium of existing blood vessels is activated by VEGF, and phenotypic switching from tip cell to stalk cells occurs through the mechanism described. VEGF binds to VEGFR2 and activates EC to become tip cell that is situated at the migrating tip of the sprout (and hence called tip cell). VEGFR2 activation results in the expression of notch ligand DLL4. DLL4 activates notch receptors in adjacent EC, which reduces VEGFR2 expression and thus decreases sensitivity to VEGF. This loss of VEGF sensitivity in EC results in loss of tip cell phenotype rather helps activate stalk cell phenotype. The equilibrium between the Jag1-notch and DLL4-notch signaling is essential for angiogenesis regulation and differentiation of stalk cell to tip cell. VEGF, vascular endothelial growth factor; VEGFR2, VEGF receptor 2; Jag1, jagged-1; DLL4, delta-like 4.

There are, furthermore, two types of angiogenic processes that occur in humans: intussusceptive angiogenesis and sprouting (Patan, 2010). The former is characterized by a splitting process in which the wall of an already formed vessel extends into the lumen, thereby causing a single vessel to split into two. This process predominately occurs during embryonic life. In contrast, sprouting angiogenesis is characterized by sprouts of ECs growing toward a pro-angiogenic stimulus [i.e., VEGF or fibroblast growth factor (FGF)]. In this type of angiogenesis, an endothelial tip cell secretes proteolytic enzymes from its filopodia in order to digest and open a path through the extracellular matrix (ECM) toward a VEGF stimulus. The process by which an EC becomes designated as a tip cell is controlled by the VEGF-Delta like ligand 4 (Dll4)-Notch signaling cascade (Leslie et al., 2007). Briefly, this process determines that the cell with the highest VEGF concentration is delegated as the tip cell. Behind the tip cell is a collection of stalk cells that proliferate as they follow behind the VEGF drawn tip cell, thus causing the capillary sprout to elongate (Figure 6) (Benedito et al., 2009; De Groot et al., 2010; Siemerink et al., 2013; Pedrosa et al., 2015; Antfolk et al., 2017). In addition, the stalk cells attract pericytes that provide support to ECs by depositing basement membrane. As the vessel elongates, intracellular vacuoles develop and coalesce, forming a lumen within the chain of stalk cells. Thus, a new vessel lined with quiescent phalanx cells (non-proliferating cells) provides a pathway to re-oxygenize and replenish a previously hypoxic tissue. In addition, embryonic angiogenesis has also been linked to lysophosphatidic acid (LPA) metabolism, a potent lipid mediator with various biological functions. LPA is said to activate the transcription regulators YAP and TAZ, which promote sprouting angiogenesis through downregulation of anti-angiogenic Dll4 expression (Yasuda et al., 2019). Insufficient angiogenesis is linked to the pathogenesis of many diseases such as ischemia, which can result in MI, stroke, and atherosclerosis. In contrast, EC hyper-proliferation can lead to cancer, pulmonary artery hypertension (PAH), and many inflammatory-related diseases.

For instance, increased ROS levels can contribute to endothelial dysfunction, which can result in activation of inflammatory responses and lead to atherosclerosis. This can occur due to the effects of ROS in reducing NO bioavailability by uncoupling the eNOS-catalyzed reduction of oxygen from the oxidation of L-arginine, thereby stimulating the production of ONOO- and causing the NO-producing enzyme to become a ROS-producing enzyme (Schulz et al., 2011). In addition, ROS production leads to a disruption of the glycocalyx, activation of myeloid and progenitor stem cells, and eventual accumulation of vascular smooth muscle-like cells within the intima, hence promoting intimal thickening. Upon exposure to hypercholesterolemia, these cells accumulate oxidized LDL and are hence termed “foam cells.” Hence, it is quite clear that the role of ROS is almost essential in the development of atherosclerosis (Burtenshaw et al., 2019).

Furthermore, capillaries have variable permeability depending on their type. For example, it could be either fenestrated, sinusoidal, or continuous (Risau, 1998). Most capillaries found in the body are of the continuous type, which have low permeability; they can be found in skeletal muscle, heart muscles, central nervous system, and dermis. Fenestrated capillaries are of relatively higher permeability and are usually found in the small intestine, endocrine glands, and kidneys. Sinusoidal capillaries have very large inter-cellular clefts and, hence, have the highest permeability. They can be either venous sinusoids (exclusively found in the bone marrow, spleen, and liver) or lymphatic sinusoids in lymph nodes.

The angiogenic potential of ECs derived from the heart has been shown to be the highest out of all organ systems in the body (Marcu et al., 2018). This capacity is due to enhanced expression of angiogenic genes and signaling factors, including upregulation of transcription factors like zinc fingers, T-box, and basic-helix-loop-helix as well as signaling factors such as EBF3, NCOA7, PRRX1, and RARB. These factors distinguish heart ECs from other ECs in the body as they are especially unique and mostly exclusive to the heart. In support of their high angiogenic potential, ECs found in the heart also have the highest rates of metabolism, demonstrated by their high rates of oxygen consumption and glycolysis (Peters et al., 2009). Non-heart ECs also perform glycolysis for ATP production but at significantly lower rates when compared to heart ECs (Peters et al., 2009).

## Carbohydrate Metabolism in ECs

### Mitochondrial Oxidative Phosphorylation vs. Glycolysis

**Figure 7:**
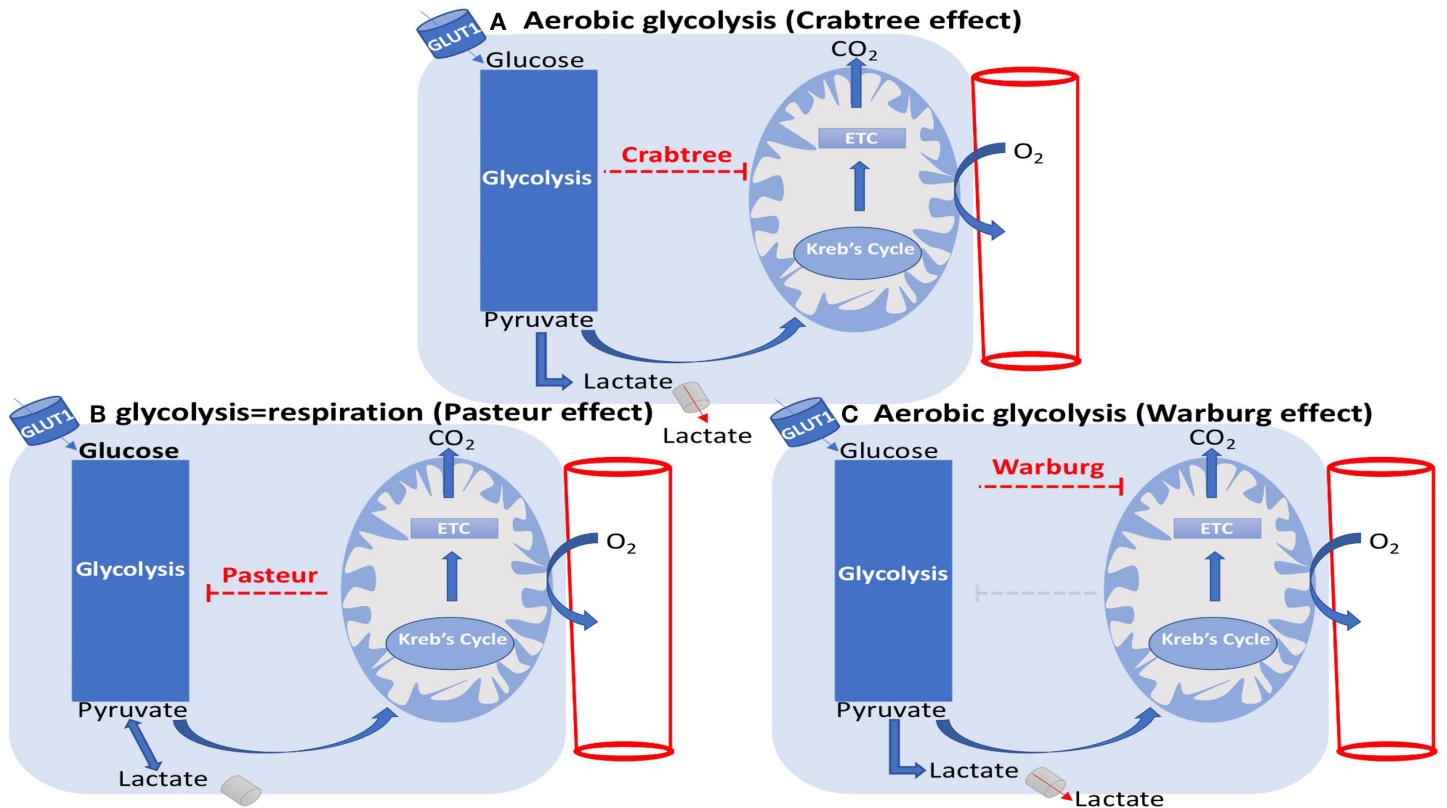
EC metabolism: Crabtree, Pasteur, and Warburg effects. **(A)** Aerobic glycolysis (Crabtree effect). **(B)** glycolysis=respiration (Pasteur effect). **(C)** Aerobic glycolysis (Warburg effect). **(A)** The Crabtree effect is the enhancement of glycolysis and suppression of mitochondrial respiration, which results in lactate generation. **(B)** The Pasteur effect is the enhancement of mitochondrial respiration and inhibition of glycolysis. **(C)** The Warburg effect is the inhibition of oxidative phosphorylation, leading to lactate generation in spite of the presence of oxygen.

It can be said that ECs are unselfish in the sense that although they have direct access to oxygen from the RBCs flowing within their lumens, they require low oxygen levels and depend on adenosine triphosphate (ATP) yielded by glycolysis as their main energy source for survival and proliferation (Krützfeldt et al., 1990; Culic et al., 1997; Quintero et al., 2006; De Bock et al., 2013a). Contrary to other cell types where mitochondrial aerobic oxidative phosphorylation is considered as the primary source of energy (De Bock et al., 2013a), ECs demonstrate Warburg effect by relying on glycolysis while being in an oxygen-rich environment (Krützfeldt et al., 1990; Culic et al., 1997; De Bock et al., 2013a). Following glycolysis, cells must choose between aerobic or anaerobic pathways to continue respiration (Figure 7). This decision, however, is made based on the status of the cell. Since ECs have low demand for oxygen, they follow the anaerobic pathway where pyruvate is converted to lactic acid by lactic acid dehydrogenase (Phypers and Pierce, 2006). In other cells that use the aerobic pathway, pyruvate travels to the matrix of the mitochondria to enter the Krebs cycle followed by the electron transport chain.

ECs additionally decrease their oxygen consumption rate (OCR) when the concentration of glucose is above 0.1 mM, demonstrating the Crabtree effect (Crabtree, 1929), where glycolytic ATP generation rate is high compared to the mitochondrial content (Koobs, 1972). In other words, the Crabtree effect is the suppression of mitochondrial oxidative phosphorylation by glycolysis as observed in ECs. The mirror image of the Crabtree effect is the Pasteur effect where mitochondrial oxidative phosphorylation inhibits glycolysis. The inhibition or failure of the Pasteur effect represents the Warburg effect (Figure 7) (Barros et al., 2020). In the case of hyperglycemia, where the excess of glucose is more than the capacity of the glycolytic pathway, glucose will enter a two-step pathway termed the polyol pathway. Here, glucose is reduced to sorbitol by the enzyme aldose reductase; sorbitol is then converted to fructose (Lorenzi, 2007; Tang et al., 2012). As previously mentioned, ECs greatly rely on glycolysis to the extent that over 80% of their energy is produced through this mode (Fitzgerald et al., 2018). Glycolysis is an adaptive mechanism developed by the body to quickly vascularize the tissue without consuming too much oxygen. This importance is apparent in hypoxic areas where the EC uses anaerobic glycolysis to maintain low oxygen consumption, thereby preserving oxygen for the surrounding perivascular cells.

Additional benefits of ECs utilizing glycolysis is that they minimize the production of ROS and generate ATP faster than could be achieved by oxidative phosphorylation. It has also been shown that EC glycolytic pathways are essential for proliferation and angiogenesis. Blockage of the essential glycolytic enzyme PFKFB3 impaired not only EC proliferation but also migration. Interestingly, EC sprouting and migration were enhanced when mitochondrial oxidative phosphorylation was inhibited (De Bock et al., 2013a; NCBI, 2020). Furthermore, it was shown that low levels of PFKFB3, and hence glycolysis, shifted the tip:stalk cell phenotype ratio in favor of the stalk cells, indicating a decrease in tip cell migration. This is a unique finding as it was previously thought that the VEGF-Dll4-Notch signaling pathway was the only controlling factor for tip:cell phenotype expression. Furthermore, this finding can be explained by the fact that tip cells have been found to be more glycolytic than stalk cells as they express higher levels of PFKB3 (Cruys et al., 2016). It has also been found that FGF, in addition to VEGF, are essential for glycolysis-controlled angiogenesis by controlling hexokinase (Hk2) expression. Pan endothelial Hk2 deletion during embryonic development significantly reduced angiogenesis as well as arterial development and branching in mice (Yu et al., 2017). When Hk2 was deleted during adult life, it resulted in significant reduction in the number of tip cells, thus reducing EC proliferation.

### ROS Formation in Carbohydrate Metabolism

**Figure 8:**
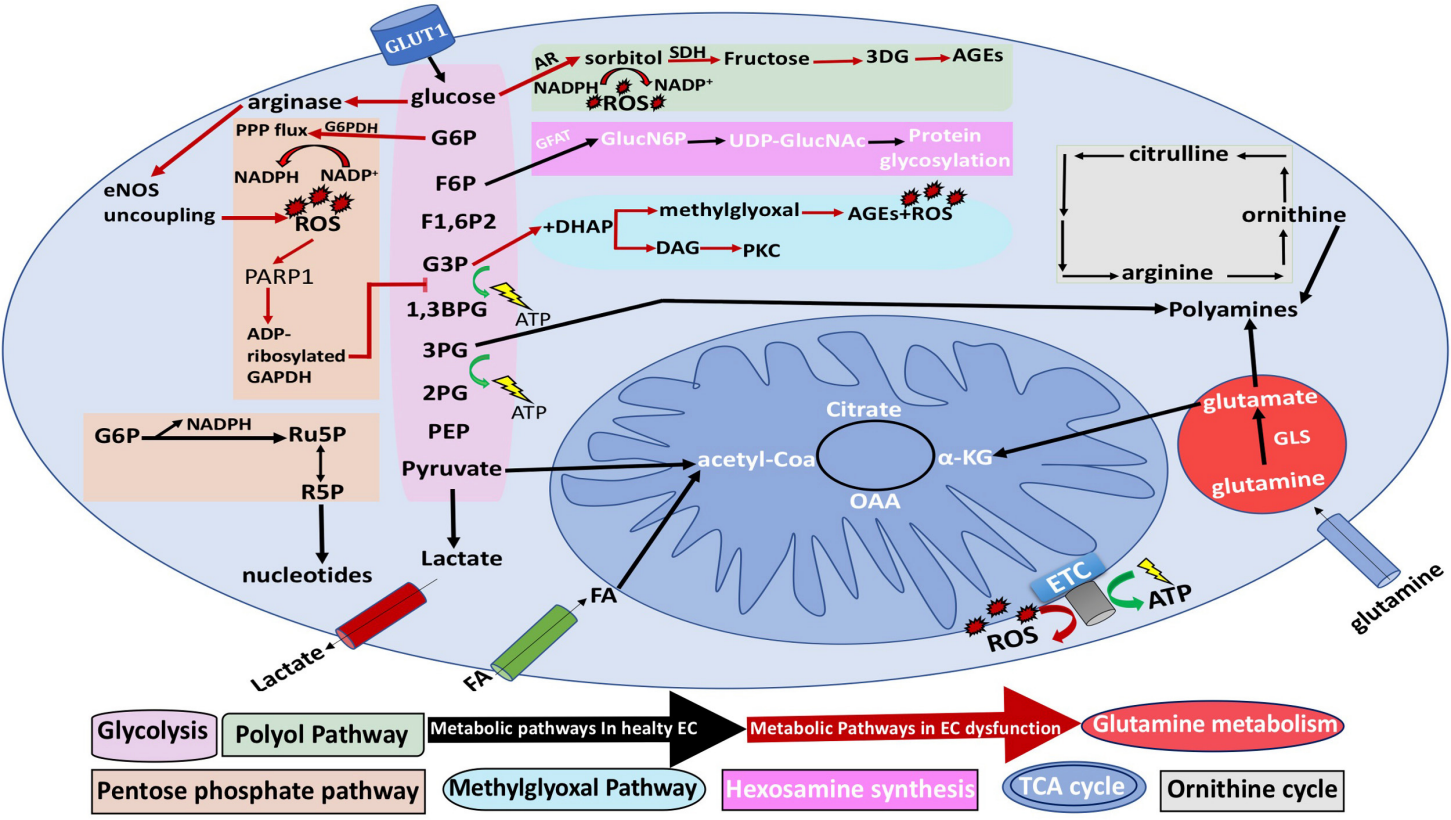
General EC metabolism and the metabolic pathways associated with EC dysfunction. Black arrows represent normal EC metabolic processes, while red arrows represent EC dysfunction. NO, nitric oxide; 3PG, 3-phosphogylcerate; 1,3BPG, 1,3-bisphosphoglycerate acid; α-KG, α-ketoglutarate; CoA, coenzyme A; ETC, electron transport chain; F1,6P2, fructose-1,6-bisphosphate; F2,6P2, fructose-2,6-bisphosphate; F6P, fructose-6-phosphate; FA, fatty acid; G3P, glyceraldehyde-3-phosphate; 2PG, 2-phosphoglyceric acid; PEP, phosphoenolpyruvate G6P, glucose-6-phosphate; GLS, glutaminase; GSH, reduced glutathione; NADPH, nicotinamide adenine dinucleotide phosphate; R5P, ribose-5-phosphate; ROS, reactive oxygen species; PARP1, polyADP-ribose polymerase 1; Ru5P, ribulose-5-phosphate; TCA, tricarboxylic acid; and UDP-GlucNAc, uridine diphosphate *N*-acetylglucosamine; AR, aldose reductase; eNOS, endothelial nitric oxide synthase; G6PDH, glucose-6-phosphate dehydrogenase; GFAT, glutamine fructose-6-phosphate amidotransferase; GlucN6P, glucosamine-6-phosphate; PKC, protein kinase C; SDH, sorbitol dehydrogenase; OAA, oxaloacetate.

Hyperglycemic states aid in increasing ROS production through several mechanisms—uncoupling of eNOS, NOX activation, and the impairment of the pentose phosphate pathway, which results in glycolytic flux suppression and the diversion of glycolytic intermediates into different pathological pathways. Through ADP-ribosylation of the glycolytic enzyme glyceraldehyde-3-phosphate dehydrogenase by polyADP-ribose polymerase, ROS and advanced glycation end product (AGEs) production will rise (Figure 8) (Eelen et al., 2015). The reduction reaction of aldose reductase can result in the depletion of NADPH stores by the conversion of NADPH to NADP+. Glutathione (GSH) is an antioxidant that is necessary for redox homeostasis and is maintained in the reduced form by NADPH. Thus, losing NADPH stores will prevent the reduction of GSH, leading to ROS accumulation. The elevated levels of aldose reductase are correlated to toxicity (De Bock et al., 2013b).

Furthermore, it has been hypothesized that glycogen stores in ECs are used as a backup source of energy when ECs migrate to regions with low glucose concentration (Bierhansl et al., 2017). Although ECs do not have a high demand on energy. EC mitochondria are thought to have an essential role in signaling pathways and in countering stressful events (Dranka et al., 2010). Hyperglycemia will lead to overproduction of mitochondrial ROS in ECs through glucose shunting into the hexosamine biosynthetic pathway (HBP) in association with the activation of pro-inflammatory signaling processes (Stefano et al., 2016). Moreover, HBP contributes to the functionality of proteins in ECs by yielding its major end product uridine diphosphate N-acetylglucosamine (UDP-GlcNAc). UDP-GlcNAc is required for protein N- and O-linked glycosylation, which is crucial for proteins to function (Ngoh et al., 2010). Physiologically, only 2–5% of glucose enters the HBP (Hart et al., 2007; Peterson and Hart, 2016). Glutamine fructose-6-phosphate aminotransferase (GFAT) is the rate-limiting enzyme of HBP, which forms glucosamine-6-phosphate by using glutamine and fructose-6-phosphate. Consequently, UDP-GlcNAc is generated as a result of a combination of acetyl-CoA, ATP, and glucosamine-6-phosphate (Mapanga and Essop, 2016; Willems et al., 2016).

In a high glucose state, ROS mediates the expression of VEGF C. The lysophosphatidic acid 1/3 (LPA 1/3) will enhance aerobic glycolysis in hyperglycemia (Huang et al., 2018). Interestingly, the peroxisome proliferator-activated receptor-γ coactivator 1α (PGC-1α) assists in maintaining cell survival by reprogramming EC metabolism. PGC-1α also enhances the energy metabolism of mitochondria and the cellular extent of ROS detoxification (Li et al., 2017). The relationship between ROS and carbohydrate metabolism ECs is one that is truly self-propagating and bidirectional. Not only does glucose metabolism in ECs lead to ROS production, but newly generated ROS also enhance further glucose uptake, leading to a seemingly unabated cycle of ROS production.

Naturally, the body has several antioxidant mechanisms to reduce the consequences of the intense ROS production, but in pathological conditions such as hypoxia-induced ROS production, these constraints can be lost and highlight the need for further research in this area. The effects of glucose metabolism on ROS production can be studied through observing the effects of hyper- and hypoglycemia on ROS production. Glucose metabolism-induced ROS can be of either cytosolic or mitochondrial origin (de Zeeuw et al., 2015). A study showed that an increased level of glucose (400 mg/dl) administration for 3 days had a significant effect in increasing ROS production compared to the amount of ROS formed after exposure to low levels of glucose (100 mg/dl) for the same period of time. Additionally, a protein kinase C (PKC) activator, such as phorbol myristic acid (PMA), also increases ROS. This is also supported by *in vitro* studies showing that ECs deprived of glucose or with inhibition of mitochondrial pyruvate transport had reduced production of ROS as compared to ECs not subjected to such intervention (Galloway and a, 2012).

Mitochondrial ROS generation through carbohydrate metabolism can occur through many proteins such as complex I (CI), complex II (CII) pyruvate dehydrogenase, monoamine oxidases, and cytochrome c (Figure 8) (Liemburg-Apers et al., 2015). CI and CIII are involved in the ETC and are characterized mostly by their ability for ROS generation. In CI, ROS production can occur at two sites: the flavin mononucleotide (FMN) site and the iron–sulfur (Fe–S) site. In CIII, ROS can be produced at the quinol-oxidizing site (Qo). It has been shown previously that if ROS levels exceed a certain threshold and exceed the EC antioxidant capabilities, they can lead to oxidative stress-induced cell damage. This can occur through the free radicals interacting with protein Fe–S complexes resulting in the generation of hydroxyl radicals that are highly reactive and can damage essential cellular components such as lipids, proteins, and DNA. In fact, the ROS can even react with the Fe–S complexes present in CI, causing their overoxidation and resulting in the irreversible deactivation of CI, ultimately impairing oxidative phosphorylation (Liemburg-Apers et al., 2015). One might theorize that the impairment of oxidative phosphorylation would lead to a decrease in ROS production since the main ROS generating enzymes, like CI and CIII, are no longer functioning, but in fact, this inhibition actually stimulates further ROS generation. This paradox is due to the fact that during oxidative stress, there is increased glucose uptake and glycolysis (Liemburg-Apers et al., 2015), thereby further stimulating glycolysis and overworking the remaining functional enzymes involved in oxidative phosphorylation, ultimately resulting in more ROS production.

When oxygen levels are normal, the oxygen domains of HIF-1a are hydroxylated by prolyl-4-hydroxylates (PHDs); this hydroxylation is a signal that tags the protein with ubiquitin to mark for proteasomal degradation of HIF-1a (Strowitzki et al., 2019). During hypoxic conditions, there is no hydroxylation of HIF-1a protein by PHD, hence allowing HIF-1a to interact with HIF-1b, which will drive increased transcription of GLUT1 genes. In physiologic conditions, ECs increase glucose uptake through glucose transporter (GLUT) transport during oxidative stress to allow glucose to go through the pentose phosphate pathway and produce increased NADPH and GSH levels, which can play a role in ROS scavenging (Liemburg-Apers et al., 2015). However, excess oxidative stress can lead to excessive glucose uptake into the EC via GLUTs, leading to an accumulation of oxidative phosphorylation substrates and elevated ratios of NADH/NAD and FADH/FAD; this could cause reduction of the CI and CIII complexes and increased electron leakage, ultimately leading to free radical production (Turrens et al., 1985; Kussmaul and Hirst, 2006).

## Lipid Metabolism and ECs

### Fatty Acid Synthesis

Lipids can be synthesized in our bodies, a process termed lipogenesis, where lymphatic ECs regulate lacteal-mediated uptake of intestinal lipids (Adams and Alitalo, 2007). The synthesis of lipids requires NADPH availability (Chon et al., 2017). Furthermore, the *de novo* lipid synthesis mediated by fatty acid synthase (FAS) is critical to preserving eNOS activity, membrane association, and palmitoylation or the covalent attachment of FAs (Wei et al., 2011). FAs have two paths with different outcomes; they can be used as an energy source in catabolic processes or can be esterified and stored. The excess lipids are stored in specialized intracellular organelles known as lipid droplets (LDs). LDs contain sterol esters and triglycerides, which act as energy reservoirs (Thiam et al., 2013). FAs will be incorporated into LDs, whenever they are in excess (Majzner et al., 2016). Moreover, ECs have LDs and express hormone-sensitive lipase, an important enzyme for triglyceride breakdown from LDs (Harjes et al., 2017). Tunneling nanotubes (TNTs) form thin membrane bridges to connect the distant ECs together and enable them to transmit signals and exchange ions, organelles, and proteins. Interestingly, VEGF enhances the transport of LDs through TNTs and, more importantly, it was shown that LDs are a cargo of TNTs (Astanina et al., 2015).

**Figure 9:**
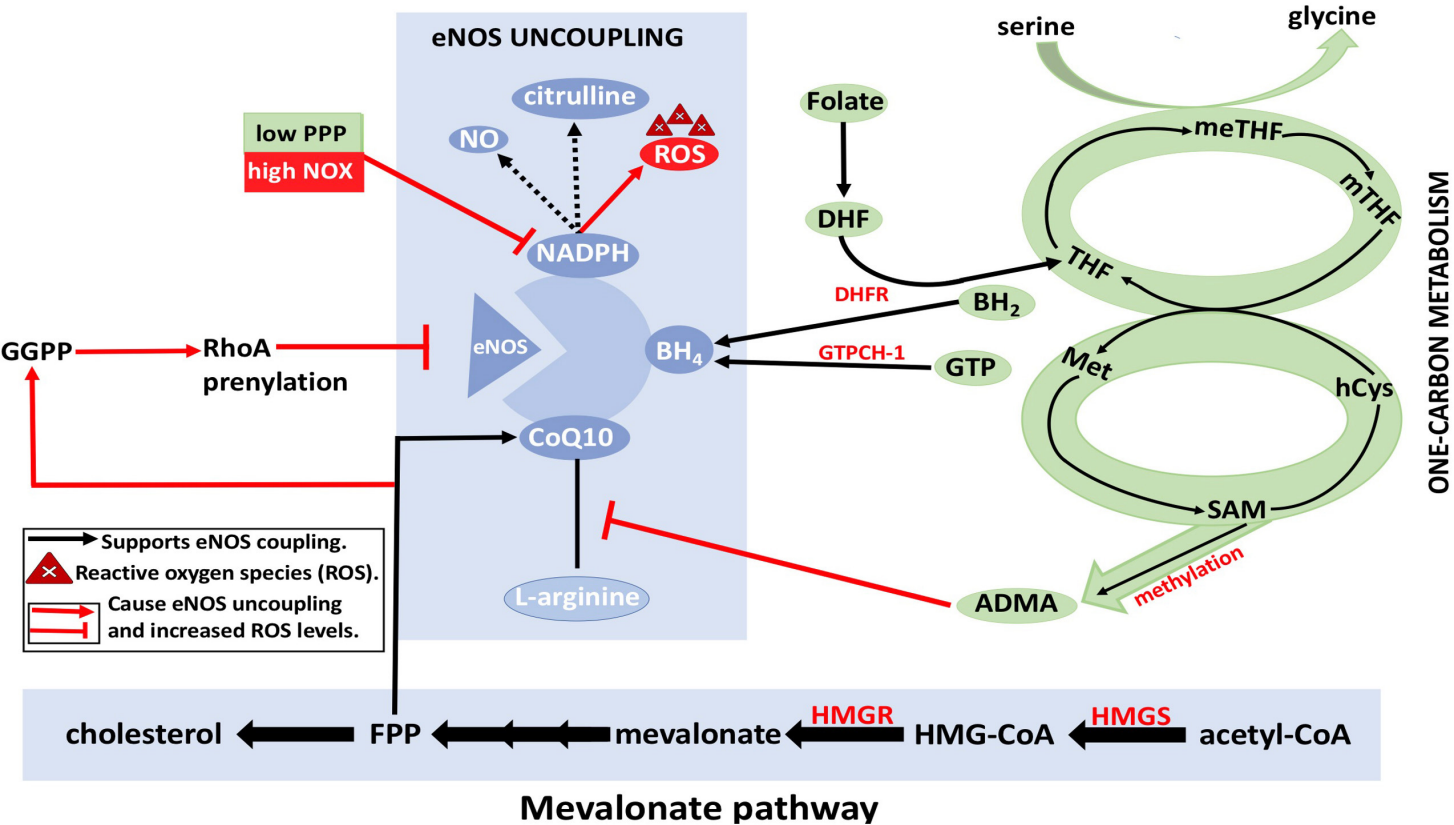
eNOS uncoupling and Dysregulation of EC metabolism. BH_4_, NADPH, and CoQ10 are essential cofactors for eNOS coupling and synthesis of nitric oxide (NO). With the reduction in the levels of arginine and/or folate, BH4 may result in eNOS uncoupling, which in turn results in ROS formation by eNOS (instead of NO). This eventually leads to disruption of EC metabolism. Reduction in arginine reduces formation of citrulline, which results in electron that is released from NADPH to be transferred to O_2_ molecules resulting in superoxide (${\text{O}}_{2}^{•-}$) formation. Furthermore, asymmetric dimethylarginine (ADMA) causes a reduction in eNOS activity by competing with arginine for eNOS binding. ADMA synthesis from methylation of L-arginine by methyldonor S-adenosylmethionine (SAM) in the one-carbon metabolism is shown (in green). RhoA also plays a role in eNOS inhibition by degrading eNOS mRNA transcripts. Geranylgeranyl pyrophosphate (GGPP) and farnesyl pyrophosphate (FPP) from the mevalonate pathway (in yellow) are needed for RhoA prenylation. HMG-CoA, hydroxymethylglutaryl coenzyme A; HMGR, 3-hydroxy-3-methylglutaryl-CoA reductase; MET, methionine; MTHFR, methylenetetrahydrofolate reductase; SMA, S-adenosylmethionine; C0Q10, coenzyme Q10; ADMA, asymmetrical dimethyl arginine; NADPH, nicotinamide adenine dinucleotide phosphate; THF, tetrahydrofolate; PPP, pentose phosphate pathway; meTHF; 5,10-methylene-methyltetrahydrofolate; GTPCH, GTP cyclohydrolase; BH4, tetrahydrobiopterin; FPP, farnesylpyrophosphate; NOX, NADPH oxidase; Acetyl-CoA, acetyl-coenzyme A; hCYS, homocysteine; mTHF, 5 methyltetrahydrofolate; DHF, dihydrofolate; GGPP, geranylgeranyl pyrophosphate; HMGS, 3-hydroxy-3-methylglutaryl-CoA synthase; DHFR, dihydrofolate reductase; BH2, 7,8dihydrobiopterin; NO, nitric oxide; eNOS, endothelial nitric oxide synthase.

It is unknown if the synthesized endothelial FA is forming lipids to contribute to the signaling process or to participate in membrane production. However, it has been shown that the silencing of FA synthesis resulted in eNOS membrane localization by posttranslational palmitoylation reduction. Consequently, the permeability of EC increased while sprouting phenomena were affected (Wei et al., 2011). Moreover, the formation of fibrous elements and lipid accumulation in large arteries represents atherosclerosis (Lusis, 2000). In atherosclerosis, ROS levels will be increased due to eNOS uncoupling. The dysregulation of metabolic pathways and methylation of arginine by methyldonor S-adenosylmethionine (SAM) are the underlying causes of eNOS uncoupling, which subsequently decreases the levels of eNOS coupling cofactors BH_4_ and NADPH (Figure 9) (Pircher et al., 2016; Eelen et al., 2018). Furthermore, the oxidation of lipids by ROS produces pro-angiogenic species, such as ω-carboxyethylpyrrole, that promote angiogenesis; this mechanism is independent of VEGF (Eelen et al., 2018).

### Fatty Acid Beta Oxidation and Angiogenesis

**Figure 10:**
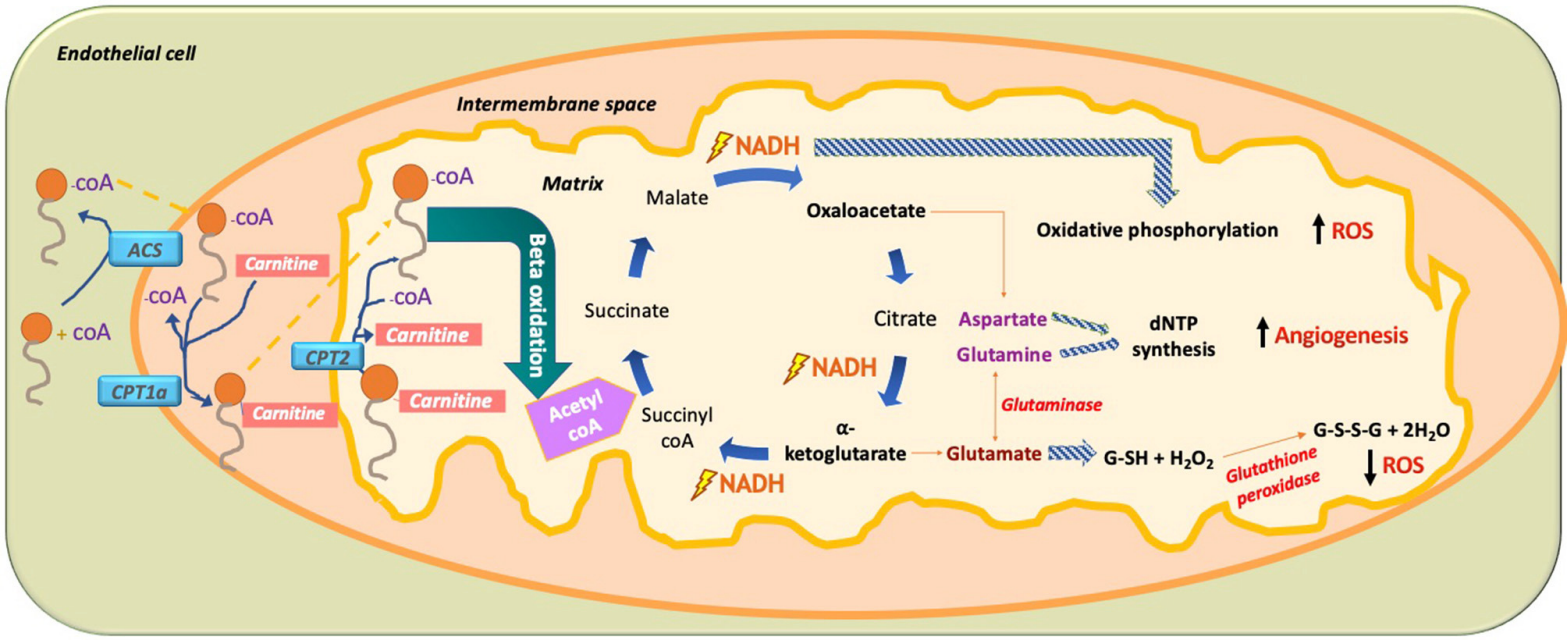
Paradoxical effect of fatty acid (FA) metabolism on ROS production in ECs. In ECs, FA become activated by combining with CoA via acetyl CoA synthetase (ACS) to produce fatty acyl CoA. With the help of mitochondrial outer membrane enzyme carbamoyl palmitoyl-transferase 1a (CPT1a), long-chain fatty acids are transported to the mitochondrial intermembrane space to combine with carnitine, while releasing CoA through CPT1a. This allows FA-carnitine, with the help of CPT2, to move into the matrix of the mitochondria where it again combines with CoA and dissociates from carnitine to produce fatty acyl CoA. Fatty acyl CoA then undergoes beta oxidation to produce 2-carbon acetyl CoA, which is subsequently incorporated into the TCA cycle. There, it produces two main intermediates, oxaloacetate and alpha ketoglutarate, as well as the energy substrate NADH. The paradoxical effect of FA oxidation-mediated ROS production is demonstrated when NADH used in oxidative phosphorylation increases ROS production. Amino acid (AA) glutamate derived from alpha-ketoglutarate is used to produce the hydrogen peroxide oxidizing agent glutathione (GSH), which neutralizes H_2_O_2_ and thus reduces ROS. In addition, the AA aspartate (derived from oxaloacetate) and the AA glutamine (derived from the AA glutamate through glutaminase) are used for nucleotide synthesis in EC, thus increasing DNA synthesis and increasing EC proliferation. FA, fatty acid; ACS, acetyl CoA synthetase; CPT1a, carbamoyl palmitoyl transferase 1a; CPT2, carbamoyl palmitoyl transferase 2; NADPH, nicotinamide adenine dinucleotide phosphate; dNTP, deoxynucleoside triphosphate; ROS, reactive oxygen species.

In 2015, Carmeliet et al. conducted a groundbreaking study demonstrating the role of FA beta oxidation (FAO) in EC proliferation (Schoors et al., 2015). It was found that the isotope-labeled 16 carbon FA palmitate was incorporated into the tricarboxylic acid cycle (TCA cycle). FAO contributed many more carbons to the TCA cycle than one might expect, almost reaching to the levels contributed by glucose and glutamate (Figure 10). This is interesting to note as conventionally it had been thought that glucose provided 85% of the carbons needed for the TCA cycle intermediate production, but these data demonstrate that, in fact, palmitate contributes a significant portion of the carbons needed to make the TCA cycle intermediates. It should be noted that fatty acid oxidation only contributes 5% of the total ATP levels in ECs; thus, one can conclude that the FA carbons being incorporated into TCA cycle intermediates are not being used for ATP production but rather for another purpose like dNTP nucleotide synthesis (Figure 10).

Knockdown of CPT1a, the enzyme that allows FA to be combined with carnitine so it can move into the mitochondrial matrix for beta oxidation, produced several effects in ECs (Schoors et al., 2015). The first and most noticeable effect was that knockdown of CPT1a significantly decreased vessel sprouting in both vessel length and amount. This effect was not due to a decrease in EC migration and motility but rather due to a decrease in EC proliferation. Rather than affecting ATP production, blockage of FA beta oxidation actually affected *de novo* nucleotide synthesis needed for DNA replication for new EC proliferation. Carbons from the labeled palmitate replenished the TCA cycle and was incorporated into the newly formed DNA of the proliferating ECs through aspartate and glutamate (pyrimidine and purine DNA nucleotide precursors), as well as through uridine monophosphate (UMP; a pyrimidine nucleoside precursor). Knockdown of CPT1a reduced the amount of palmitate-labeled carbon incorporation into new EC DNA. Even more, the final deoxyribonucleotide forms (dNTP) were all reduced for both pyrimidines (dCTP, dTTP) and purines (dATP, dGTP). Interestingly, when the CPT1a-blocked ECs were given acetate, which was converted into acetyl CoA, dNTP levels were restored and the angiogenic defect was rescued. Since FA metabolism works alongside glucose metabolism for dNTP synthesis, it was found that inhibition of FAO slightly increased glucose oxidation suggesting a partial compensatory mechanism; however, it was still insufficient to rescue deficient dNTP synthesis and EC proliferation. Although FAs were found to be irreplaceable for DNA synthesis; compensatory pathways restored RNA and protein synthesis. Although Carmeliet et al. were the first to elucidate that FA has a direct link to dNTP synthesis in ECs (Schoors et al., 2015), a study done in 1988 (Hülsmann and Dubelaar, 1988) also revealed similar outcomes of an increase in tagged oleate FA carbons being incorporated into DNA following beta oxidation of ECs. Another study in 1987 found that in cow ECs, there was high activity of CPT1a; however, the investigators attributed that FAO played a role in ATP generation and did not suggest any role of this enzyme in EC proliferation (Leighton et al., 1987). Overall, FAO is important for endothelial stalk cell proliferation.

### Paradoxical Effect of Fatty Acid Beta Oxidation in ROS Production

Blocking of CPT1a in Carmeliet's study (Schoors et al., 2015) increased the amount of ROS by 20%; however, this amount did not disturb GSH or redox homeostasis levels and hence DNA synthesis was unaffected for newly proliferating ECs, ultimately suggesting that ROS did not play a role in the angiogenic potential of ECs. A study done by Pike et al. showed that in neuroglioblastoma cells, blocking of FAO by etoximir caused a decrease in NADPH and GSH levels, thus making the cells more vulnerable to oxidative stress (Pike et al., 2011). Since NADPH and GSH synthesis are linked to FAO, blockage of FAO could increase ROS (Pike et al., 2011). On the other hand, it has been shown that FAO also increases the amount of ROS levels. One reason for this finding could be the incorporation of FAO products like acetyl CoA into the TCA cycle, which has the ROS-generating enzyme a-ketoglutarate dehydrogenase. Also, it has been shown that oxidation of acyl-CoAs slightly activate Reverse Electron Transport (RET)-dependent superoxide generation in muscle fiber cells, which could also hold true for EVs (Seifert et al., 2010). These two findings may contradict each other, demonstrating a paradoxical effect of FA beta oxidation in ROS production (Figure 10). It should be noted that according to Mariana et al., the amount of ROS produced by beta oxidation in ECs is 30% increased, which is slightly more than the amount produced by FAO inhibition. Thus, FAO produces more ROS than FA inhibition (Rosca et al., 2012).

The role of fatty acids in cardiovascular pathology is a field of research that has been extensively studied. Fatty acids are known to contribute to many CVDs in the endothelium such as atherosclerosis through ROS production. It has been suggested that increased fatty acid beta oxidation can produce increased levels of ROS, thus contributing to the pathogenesis of these diseases. Araki et al. found that in hypoglycemic conditions, when there is increased triglyceride breakdown and FFAs found in the bloodstream, an increase in FAO in ECs caused an increase in mitochondrial ROS levels (Kajihara et al., 2017). ECs under low glucose (LG) conditions had increased ROS production, which was completely suppressed by etomoxir, a drug blocking CPT1a, which is an FAO inhibitor. Interestingly, although there was an increase in ROS by increased FAO, the LG actually inhibited eNOS activation and caused endothelial dysfunction. This contradicts studies that show that ROS actually activate eNOS and other pro-inflammatory enzymes (Feng et al., 2010). Furthermore, Schonfeld et al. suggested that superoxide production by FFA oxidation is mostly of mitochondrial origin in non-ECs, but of NOX origins in ECs. It has been suggested that polyunsaturated FAs induce a conformational change in p47phos of the NOX complex, inducing it into a state capable of interacting with p22phox, which can thus produce ROS (Schönfeld and Wojtczak, 2008). It should be noted that this action is simply the interaction of excess FFAs interacting with NOX in the cytosol, not the generation of ROS from beta oxidation.

Glutamine is a precursor of glutamate, which is a precursor for the antioxidant enzyme GSH; it has been found that in embryonic stem cells, a decrease in glutamine led to an increase in ROS. When a mitochondrial superoxide probe was used to measure ROS in mitochondria without glutamine, the researchers found an increase in ROS. Surprisingly, however, this increase in ROS did not decrease vascular propensity of the cells when they differentiated into ECs but in fact produced ECs with greater angiogenic capacity. This suggests a correlation between ROS and angiogenic capacity of ECs (Marsboom et al., 2016). It was also found that inhibiting glutaminase caused a decrease in EC proliferation and cell cycle progression via induction of cyclin A in the G/G1 phase of EC cycle (Durante et al., 2020).

### Effects of ROS Production on Fatty Acid Beta Oxidation

There remains a lack of data on the inverse—the effect of ROS on FA metabolism in ECs. There is a conspicuous absence of studies that demonstrate effects ROS on FA metabolism in ECs. There is, however, data about the influence of ROS on FA metabolism in tumor cells, which behave similarly to ECs in terms of their energy production (i.e., both depend on non-oxidative phosphorylation pathway, namely, glycolysis). When tumor cells are deprived of ATP, they undergo loss of attachment from the ECM, which not only causes further loss of ATP but also increased ROS production. This accumulation of ROS was found to inhibit FAO and cause tumor cell apoptosis (Schafer et al., 2009). However, this is unlike ECs where FAO contributes more to dNTP synthesis than ATP production. This supports an interesting hypothesis that an increase in ROS will inhibit FAO, potentially leading to angiogenic defects in EC. Since FAO is increased in a hypoglycemic state, angiogenesis may also be increased due to compensatory VEGF expression. As a result, increased FAO may lead to increased ROS production that then has a self-limiting effect on its own production by negative feedback. Although EC FAO is more dependent on *de novo* pathway, it is interesting to note that for the salvage pathway, an increase in ROS reduces transport of adenosine into ECs resulting in decreased salvage production of dATP nucletides (Suzuki et al., 2010).

## ROS Metabolism and Epigenetics

Epigenetics refer to all the changes in the nuclear and mitochondrial DNA, including structural and conformational changes with preserved DNA/RNA sequence. Post-translational histone modifications, DNA methylation, non-coding RNA transcripts, and ATP-dependent alterations to chromatin are the most commonly associated epigenetic alterations (Li et al., 2007; Holoch and Moazed, 2015; Allis and Jenuwein, 2016). Nowadays, epigenetic modifications are believed to be as dynamic a process as transcription. Thus, they might present a crucial link between ROS actions and CVDs. Interestingly, the epigenetic landscape is modulated by ROS and it contributes to CVD pathogenesis (Kietzmann et al., 2017). On the other hand, the levels of cellular ROS can be epigenetically regulated through various mechanisms (Castegna et al., 2015; Manea et al., 2015; Mikhed et al., 2015). Furthermore, ROS can directly modify DNA bases. For example, hydroxyl radicals can convert 5mC to 5hmC by H-atom abstraction from the methyl group (Madugundu et al., 2014). 5hmC has the ability to block methylation by interfering with DNMT1, resulting in an indirect demethylation (Branco et al., 2012). It has been suggested that superoxide can mediate cytosine methylation directly by C5 deprotonating, which is followed by a direct methyl group transfer from SAM without the need of a DNMT (Afanasev, 2014).

The programming of epigenetic includes modifications in DNA packing density that is regulated by lysine and arginine acetylation status. The previously mentioned amino acids are both positively charged and are located in the histone tails. Moreover, p300 is an example of acetyl transferases that has the ability of neutralizing the positively charged amino acids through the conversion of amines into amides, which subsequently loosens the binding of histone to DNA and eventually result in easier transcription. On the other hand, transcription is inhibited by the action of histone deacetylases (HDACs), in which acetyl groups are removed and histone-DNA binding gets tight. Interestingly, out of the four main classes of HDACs, two appeared to use cysteine oxidation directly for redox regulation. Also, HDAC4 turned out to have an important role in ECs by maintaining autophagy and the expression of VEGFR2 as well as enhancing angiotensin II-induced inflammation in ECs. HDAC4 overexpression in ECs results in a significant reduction in tube formation. However, in the presence of overexpressed Nox4, the HDAC4 will get phosphorylated and this will disrupt it from binding to myocyte enhancer factor-2 (Mef2A), allowing the pro-angiogenic transcription factor Mef2A to be active and free, which aids in tube formation and angiogenesis. This mechanism highlights the important effects of metabolic and redox status in the permanent changes that ECs undergo in their epigenetic programming (Narayanan et al., 2018; Schader et al., 2020).

Sirtuin-1 (SIRT1) is a class III HDAC that is NAD+ dependent. SIRT1 has multiple important functions such as regulating energy metabolism, anti-atherogenic effect, and anti-oxidant. SIRT1 mediates the acetylation of various functional proteins and is considered to be an essential longevity gene. Furthermore, SIRT1 deacetylation in endothelium and macrophages and hepatocytes is increased by hydrogen sulfide (H2S) and cystathionine γ-lyase (CSE) treatment. In addition to promoting SIRT1 deacetylation, CSE and H2S are also responsible for enhancing its stability. SIRT1 deacetylation is followed by the deacetylation of its targeted proteins (P65, P53), which reduces EC inflammation, suppresses the *de novo* synthesis of cholesterol in the liver, and inhibits macrophage cholesterol uptake (Du et al., 2019).

## Discussion

The complexity of EC metabolism is represented by the various mechanisms through which it utilizes carbohydrates, fatty acids, and amino acids as important sources for its energy and nucleotides synthesis. Interestingly, the transition from quiescent state to angiogenic state in EC (tip cell vs. stalk cell) depends on VEGF-Dll4-Notch signaling cascade and metabolic pathways that involve glucose, fatty acids, and glutamine (Marcu et al., 2018). Moreover, despite the fact that ECs line the innermost layer of blood vessels and have direct access to oxygen in the blood, ECs rely on anaerobic respiration via glycolysis for over 85% of their energy production (Fitzgerald et al., 2018). This reliance has been shown to be beneficial due to the reduction of ROS levels, faster generation of ATP, and preservation of oxygen in comparison to energy generated from oxidative phosphorylation. Furthermore, NOX are proven to be the primary source of ROS formation in ECs, which is further supported by the findings of small volume of total mitochondria to EC mass ratio. ECs depend on FAO for 85% of their carbon source (Schoors et al., 2015). On the other hand, carbohydrate metabolism and ROS share a bidirectional relationship where glucose metabolism in ECs leads to ROS production, and the newly generated ROS enhance further glucose uptake. Altogether, this loop results in an unabated cycle of ROS production that in turn may promote angiogenesis in the short term (Shafique et al., 2017).

In conclusion, ROS demonstrate paradoxical effects on EC metabolism and require further study to better understand the complex relationship between redox signaling and EC metabolism and the subsequent functional effects that modulate EC proliferation, migration, and angiogenesis. Elucidation of subcellular differences of ROS content and metabolic state between EC tip, stalk, and phalanx cells will certainly contribute to a better understanding of sprouting angiogenesis. ECs are a heterogeneous population in more ways than one due to their local environment in various organ beds and vessel types; thus, establishing “normal” levels of ROS and metabolites in these tissue-specific and/or vascular bed-specific populations of EC will pave the way for maintenance of homeostasis and prevention of CVD. The increasing prevalence of metabolic syndrome and their direct contribution to CVD underscore the importance of comparative metabolic profiling and analysis of corresponding EC phenotype in health and disease. Furthermore, as noted in this review, since tumor cell metabolism holds interesting similarities to that of ECs regarding glycolysis and proliferation, tumor EC-specific studies of ROS and metabolism may help understand EC phenotypes in tumors and may provide key information to prevent tumor angiogenesis. Future studies providing mechanistic insights in redox-dependent molecular signaling pathways that modulate EC metabolism and tissue-specific phenotype will help develop therapeutic modalities for CVD and tumor angiogenesis.

## Author Contributions

RA and EH: writing manuscript, data collection, study development, and schematic designing. CK: manuscript editing, study design, and figure editing. FWS: manuscript editing and study design. MRA: concept development, study design, and manuscript editing. All authors contributed to the article and approved the submitted version.

## Conflict of Interest

The authors declare that the research was conducted in the absence of any commercial or financial relationships that could be construed as a potential conflict of interest.

## Glossary

ROS, reactive oxygen species; ECs, endothelial cells; VEGF, vascular endothelial growth factor; FAs, fatty acids; AA, amino acid; PFKFB3, phosphofructo-2-kinase/fructose-2,6-bisphosphate; CPT1a, carnitine palmitoyltransferase 1a; GLS1, glutaminase 1; FGF, fibroblast growth factor; ECM, extracellular matrix; PAH, pulmonary artery hypertension; ${\text{O}}_{2}^{•-}$, superoxide anion; H_2_O_2_, hydrogen peroxide; ^•^OH, hydroxyl radical; NOX, NADPH oxidase; AMPK, AMP-activated protein kinase; PGC-1α, PPAR gamma co-activator 1 alpha; HIF-1α, hypoxia-inducible factor 1-alpha; eNOS, endothelial nitric oxide synthase; FOXO1, Forkhead box protein O1; CaMKKβ, Ca^2+^/calmodulin-dependent protein kinase kinase-β; mTOR, mammalian target of rapamycin; AGEs, advanced glycation end product; GSH, glutathione; UDP-GlcNAc, uridine diphosphate N-acetylglucosamine; HBP, hexosamine biosynthetic pathway; GFAT, glutamine fructose-6-phosphate aminotransferase; PGC-1α, peroxisome proliferator-activated receptor-γ coactivator 1α; PKC, protein kinase C; PHDs, hydroxylated by prolyl-4-hydroxylates; GLUT, glucose transporter; PMA, phorbol myristic acid; FMN, flavin mononucleotide; Fe–S, iron–sulfur; Qo, quinol-oxidizing site; LDs, lipid droplets; TNTs, tunneling nanotubes; SAM, S-adenosylmethionine; Dll4, delta-like ligand 4; HSL, hormone sensitive lipase; PKA, protein kinase A; TCA cycle, tricarboxylic acid cycle; UMP, uridine monophosphate; dNTP, deoxyribonucleotide forms; GMPS, guanosine monophosphate synthetase; FAO, fatty acid oxidation; GO, glucose oxidation; RONS, reactive oxygen nitrogen species; ONOO^−^., peroxynitrite; HDACs, histone deacetylases; Mef2A, myocyte enhancer factor; Nrf2, nuclear factor erythroid 2-related factor 2.
